# Supplementary material for: PPAD: a deep learning architecture to predict progression of Alzheimer’s disease
Source: Bioinformatics. 2023 Jun 30;39(Suppl 1):i149–57. doi: 10.1093/bioinformatics/btad249 (PMC10311312; doi:10.1093/bioinformatics/btad249)
Supplement: btad249_Supplementary_Data [file btad249_supplementary_data.pdf]

Supplemental Table 1 List of features in ADNI data used to train the models in the first experimental setup where models were trained on 70% of ADNI data and tested on the 30% held-out ADNI data. MRI: Magnetic Resonance Imaging.

Feature name	Description	Category	Type
**CDRSB**	Clinical Dementia Rating Scale–Sum of Boxes	Cognitive performance	Longitudinal
**ADAS11**	Alzheimer's Disease Assessment Scale 11	Cognitive performance	Longitudinal
**ADAS13**	Alzheimer's Disease Assessment Scale 13	Cognitive performance	Longitudinal
**ADASQ4**	Alzheimer's Disease Assessment Scale Q4	Cognitive performance	Longitudinal
**MMSE**	Mini-Mental State Examination	Cognitive performance	Longitudinal
**RAVLT.immediate**	Rey Auditory Verbal Learning Test (Immediate recall)	Cognitive performance	Longitudinal
**RAVLT.learning**	Rey Auditory Verbal Learning Test (Learning)	Cognitive performance	Longitudinal
**RAVLT.forgetting**	Rey Auditory Verbal Learning Test (Forgetting)	Cognitive performance	Longitudinal
**RAVLT.perc.forgetting**	Rey Auditory Verbal Learning Test (Percent Forgetting)	Cognitive performance	Longitudinal
**LDELTOTAL**	The logical memory delayed recall total	Cognitive performance	Longitudinal
**TRABSCOR**	Trail Making Test-B	Cognitive performance	Longitudinal
**FAQ**	Functional Activities Questionnaire	Cognitive performance	Longitudinal
**Ventricles**	Ventricles measurement	MRI	Longitudinal
**Hippocampus**	Hippocampus measurement	MRI	Longitudinal
**WholeBrain**	Whole Brain measurement	MRI	Longitudinal
**Entorhinal**	Entorhinal cortex measurement	MRI	Longitudinal
**Fusiform**	Fusiform Gyrus	MRI	Longitudinal
**MidTemp**	Scan of the middle temporal artery	MRI	Longitudinal
**ICV**	Intracranial volume	MRI	Longitudinal
**AGE**	Age of patient	Demographic	Longitudinal
**PTGENDER**	Gender of patient	Demographic	Cross-sectional
**PTEDUCAT**	Education years of patient	Demographic	Cross-sectional
**PTETHCAT**	Ethnicity of patient	Demographic	Cross-sectional
**PTRACCAT**	Race of patient	Demographic	Cross-sectional
**APOE4**	Number of ε4 allele	Demographic	Cross-sectional

Supplemental Table 2 List of features in NACC data for the second setup, where models were trained on ADNI data and tested on NACC data.

Feature name	Corresponding feature in ADNI
**CDRSUM**	CDRSB
**NACCMMSE**	MMSE
**MEMUNITS**	LDELTOTAL
**FAQ***	FAQ
**NACCAGE**	AGE
**SEX**	PTGENDER
**EDUC**	PTEDUCAT
**HISPANIC**	PTETHCAT
**RACE**	PTRACCAT
**NACCNE4S**	APOE4

* NACC dataset does not have FAQ feature, but it has the raw data that can be used to get it based on the following equation:

FAQ = BILLS+ TAXES+ SHOPPING+ GAMES+STOVE+MEALPREP+EVENTS+PAYATTN+ REMDATES+TRAVEL

The value of each feature that contributes FAQ value has the following range (0 = Normal, 1 = Has difficulty but does by self, 2 = Requires assistance, 3 = Dependent).

Supplemental Table 3 Optimal hyperparameter values of PPAD for each scenario in all splits in the first experimental setup. For each scenario, x→1 means that the model was trained using x visits and tested the diagnosis at the next visit.

Data split	Scenario	RNN cell	Batch size	Epochs	Dropout rate	L2
**1**	2→1	Bi-GRU	4	50	0.4	$1e^{-5}$
	3→1	Bi-GRU	16	20	0.2	$1e^{-3}$
	5→1	Bi-LSTM	2	50	0.1	$1e^{-5}$
	6→1	Bi-GRU	8	40	0.2	$1e^{-5}$
**2**	2→1	GRU	16	100	0.4	$1e^{-3}$
	3→1	GRU	2	40	0.0	$1e^{-3}$
	5→1	Bi-GRU	4	20	0.0	$1e^{-3}$
	6→1	Bi-GRU	16	30	0.3	$1e^{-7}$

**3**	2→1	Bi-LSTM	32	30	0.0	$1e^{-5}$
	3→1	Bi-GRU	4	50	0.2	$1e^{-3}$
	5→1	Bi-LSTM	8	50	0.5	$1e^{-7}$
	6→1	LSTM	2	40	0.4	$1e^{-3}$

Supplemental Table 4 Optimal hyperparameter values of PPAD-AE for each scenario in all splits in the first experimental setup. For each scenario, $x \rightarrow y$ means that the model was trained using x visits and tested the diagnosis at the next y^{th} visit ahead.

**Data split**	**Scenario**	**RNN cell**	**Batch size**	**Epochs**	**Dropout rate**	**L2**
**1**	2→1	Bi-LSTM	8	40	0.1	1e-3
	2→2	GRU	4	10	0.1	1e-3
	2→3	Bi-GRU	16	30	0.1	1e-3
	2→4	Bi-GRU	4	20	0.0	1e-3
	3→1	Bi-GRU	4	10	0.1	1e-3
	3→2	Bi-GRU	2	40	0.5	1e-5
	3→3	LSTM	4	40	0.4	1e-5
	3→4	GRU	8	30	0.0	1e-3
	5→1	GRU	4	50	0.1	1e-7
	5→2	Bi-LSTM	2	20	0.0	1e-3
	5→3	GRU	2	40	0.3	1e-7
	5→4	Bi-GRU	2	30	0.0	1e-5
	6→1	GRU	4	30	0.0	1e-5
	6→2	Bi-GRU	8	30	0.1	1e-5
	6→3	LSTM	2	10	0.1	1e-3
	6→4	Bi-GRU	4	40	0.1	1e-3
**2**	2→1	Bi-LSTM	4	10	0.4	1e-7
	2→2	Bi-GRU	8	20	0.1	1e-3
	2→3	Bi-LSTM	2	30	0.1	1e-7
	2→4	Bi-GRU	32	20	0.0	1e-3
	3→1	GRU	32	100	0.3	1e-7
	3→2	Bi-GRU	32	20	0.0	1e-5

	3→3	Bi-GRU	8	40	0.2	1e-7
	3→4	GRU	2	30	0.3	1e-5
	5→1	Bi-LSTM	8	100	0.4	1e-5
	5→2	Bi-LSTM	32	100	0.2	1e-7
	5→3	Bi-GRU	16	20	0.0	1e-5
	5→4	GRU	2	50	0.2	1e-3
	6→1	Bi-GRU	2	20	0.0	1e-7
	6→2	Bi-GRU	2	100	0.4	1e-5
	6→3	LSTM	2	30	0.2	1e-7
	6→4	Bi-GRU	2	40	0.1	1e-5
**3**	2→1	Bi-LSTM	4	10	0.3	1e-5
	2→2	GRU	32	50	0.1	1e-5
	2→3	GRU	4	40	0.2	1e-5
	2→4	Bi-GRU	16	10	0.1	1e-3
	3→1	Bi-GRU	4	100	0.4	1e-5
	3→2	GRU	8	30	0.1	1e-7
	3→3	Bi-GRU	16	30	0.1	1e-7
	3→4	Bi-GRU	8	20	0.4	1e-3
	5→1	Bi-GRU	4	50	0.4	1e-7
	5→2	Bi-GRU	8	50	0.4	1e-7
	5→3	GRU	4	10	0.1	1e-7
	5→4	Bi-GRU	2	40	0.5	1e-5
	6→1	Bi-GRU	2	100	0.4	1e-5
	6→2	Bi-LSTM	4	20	0.2	1e-5
	6→3	Bi-LSTM	2	20	0.4	1e-7
	6→4	Bi-GRU	16	30	0.2	1e-5

Supplemental Table 5 Optimal hyperparameter values of PPAD for each scenario in all splits in the second experimental setup. For each scenario, x→1 means that the model was trained using x visits and tested the diagnosis at the next visit.

Scenario	RNN cell	Batch size	Epochs	Dropout rate	L2
**2→1**	GRU	2	40	0.1	$1e^{-7}$
**3→1**	Bi-GRU	32	20	0.2	$1e^{-3}$
**5→1**	Bi-LSTM	4	10	0.0	$1e^{-7}$
**6→1**	Bi-GRU	4	30	0.2	$1e^{-5}$

Supplemental Table 6 Optimal hyperparameter values of PPAD-AE for each scenario in all splits in the second experimental setup. For each scenario, x→y means that the model was trained using x visits and tested the diagnosis at the next y^{th} visit ahead.

Scenario	RNN cell	Batch size	Epochs	Dropout rate	L2
**2→1**	GRU	2	40	0.2	1e-7
**2→2**	Bi-GRU	4	20	0.2	1e-7
**2→3**	GRU	16	10	0.0	1e-7
**2→4**	Bi-GRU	8	10	0.0	1e-7
**3→1**	Bi-GRU	2	10	0.4	1e-3
**3→2**	Bi-GRU	16	20	0.1	1e-5
**3→3**	GRU	8	30	0.1	1e-7
**3→4**	GRU	16	40	0.0	1e-3
**5→1**	Bi-LSTM	2	30	0.3	1e-5
**5→2**	Bi-GRU	2	10	0.2	1e-3
**5→3**	Bi-LSTM	4	30	0.3	1e-5
**5→4**	Bi-GRU	16	10	0.0	1e-5
**6→1**	Bi-LSTM	4	30	0.1	1e-7
**6→2**	GRU	64	100	0.1	1e-7
**6→3**	GRU	8	10	0.1	1e-5
**6→4**	GRU	8	50	0.4	1e-3

Supplemental Table 7 Summary of the number of Non-converter and converter cases in each scenario in the first experimental setup.

	Train		Test	
**Scenario**	**Non-Converter**	**Converter**	**Non-Converter**	**Converter**
**2_1**	499	275	211	132
**2_2**	378	294	159	108
**2_3**	291	231	124	61
**2_4**	219	140	84	53
**3_1**	438	336	159	108
**3_2**	349	323	124	61
**3_3**	261	261	84	53
**3_4**	203	156	47	40
**5_1**	379	395	84	53
**5_2**	303	369	47	40
**5_3**	239	283	32	23
**5_4**	195	164	23	18
**6_1**	363	411	47	40
**6_2**	297	375	32	23
**6_3**	237	285	23	18
**6_4**	193	166	17	9

Supplemental Table 8 Summary of the number of Non-converter and converter cases in each scenario in the second experimental setup.

	Train		Test	
**Scenario**	**Non-Converter**	**Converter**	**Non-Converter**	**Converter**
**2_1**	745	460	1447	4671
**2_2**	569	471	869	3530
**2_3**	439	382	522	2527
**2_4**	335	233	317	1800
**3_1**	666	539	869	3530
**3_2**	516	524	522	2527
**3_3**	399	422	317	1800
**3_4**	307	261	198	1216
**5_1**	573	632	317	1800
**5_2**	448	592	198	1216
**5_3**	361	460	121	768
**5_4**	292	276	71	480
**6_1**	545	660	198	1216
**6_2**	438	602	121	768
**6_3**	356	465	71	480
**6_4**	288	280	44	262

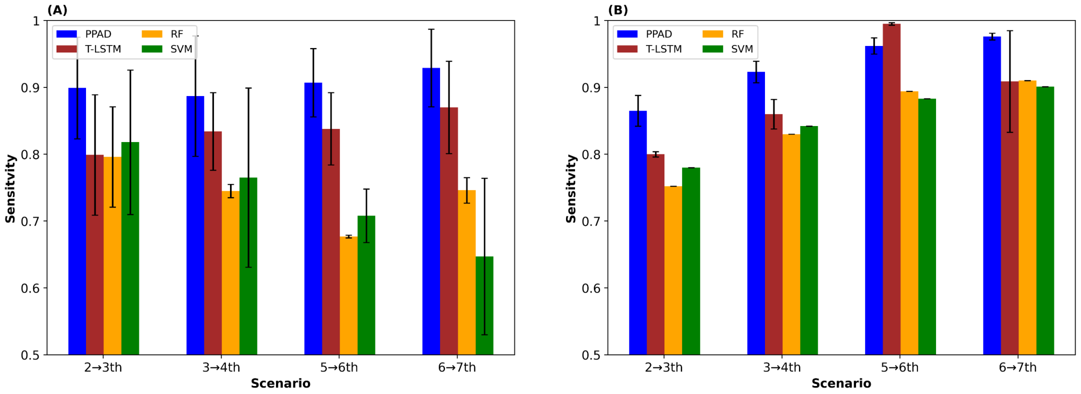

Supplemental Fig. 1 Sensitivity scores for PPAD models to predict conversion to AD at the next visit. (A) Models tested on held-out samples in ADNI after training using 2, 3, 5, and 6 visits in ADNI, respectively. (B) Models tested on NACC after training using 2, 3, 5, and 6 visits in ADNI, respectively.

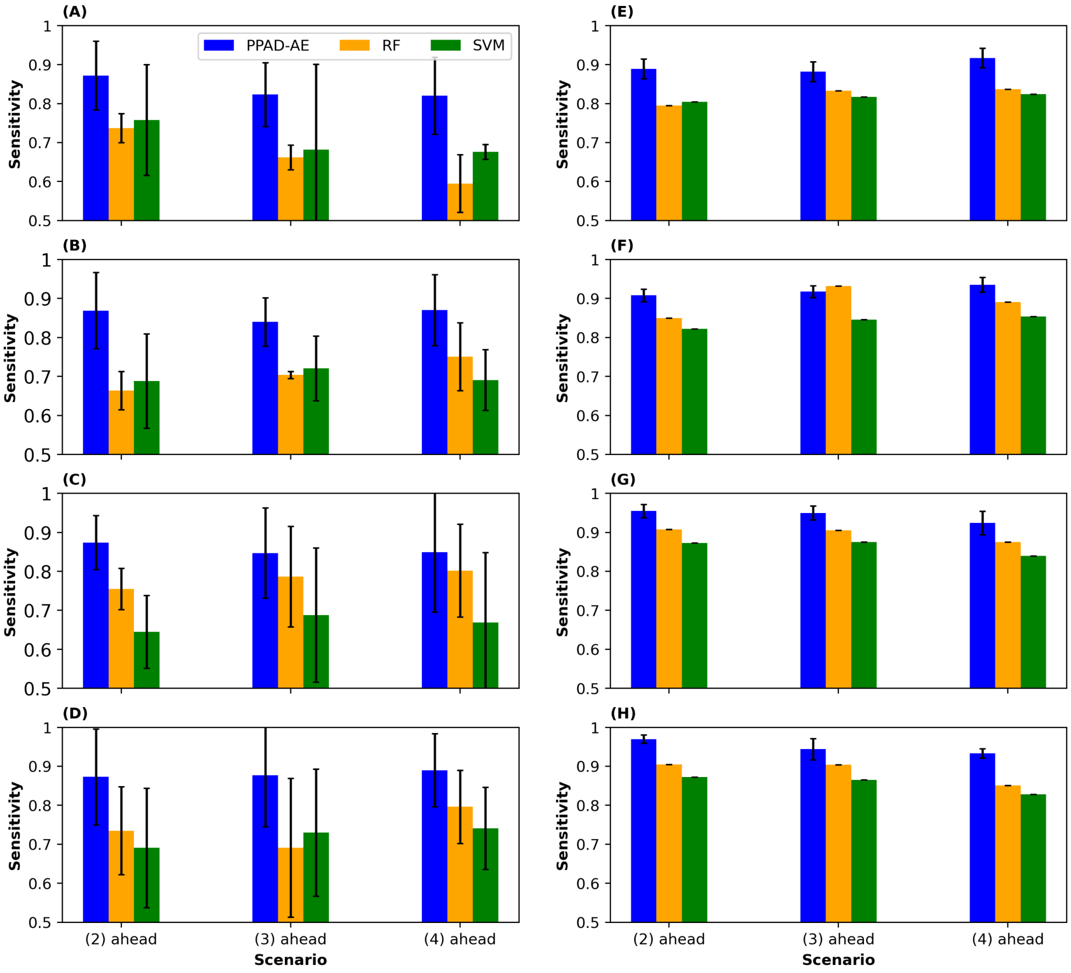

Supplemental Fig. 2 Sensitivity scores for PPAD-AE models to predict progression to AD at the next second, third, and fourth visits ahead. (A, B, C, and D) Models tested on held-out samples in ADNI after training using 2, 3, 5, and 6 visits in ADNI, respectively. (E, F, G, and H) Models tested on NACC after training using 2, 3, 5, and 6 visits in ADNI, respectively.

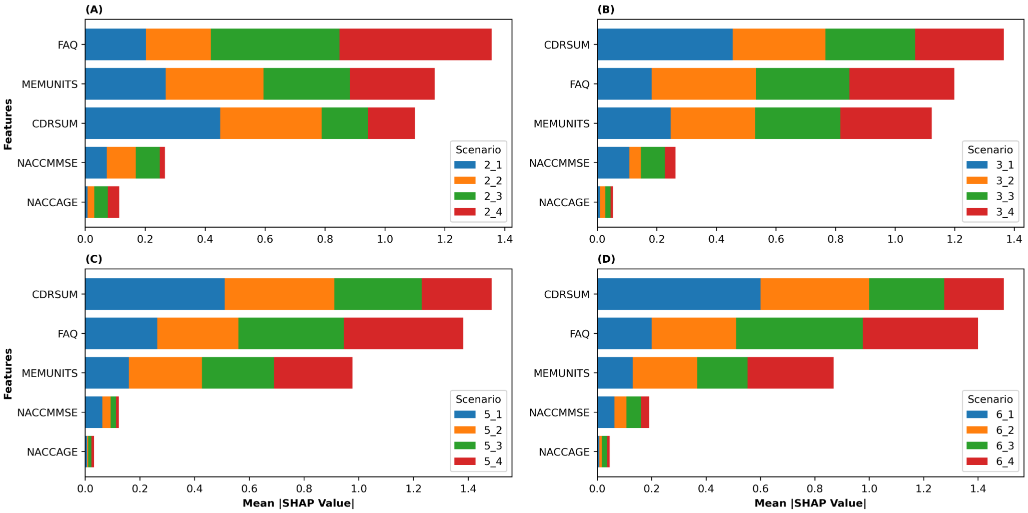

Supplemental Fig. 3 SHAP values for all features used in the second experimental setup using A) 2 B) 3 C) 5 D) 6 visits to train the models. 2_1 means trained using two visits to predict conversion to AD at the next visit, 3_2 means trained using three visits to predict conversion at two visits ahead, and so on.

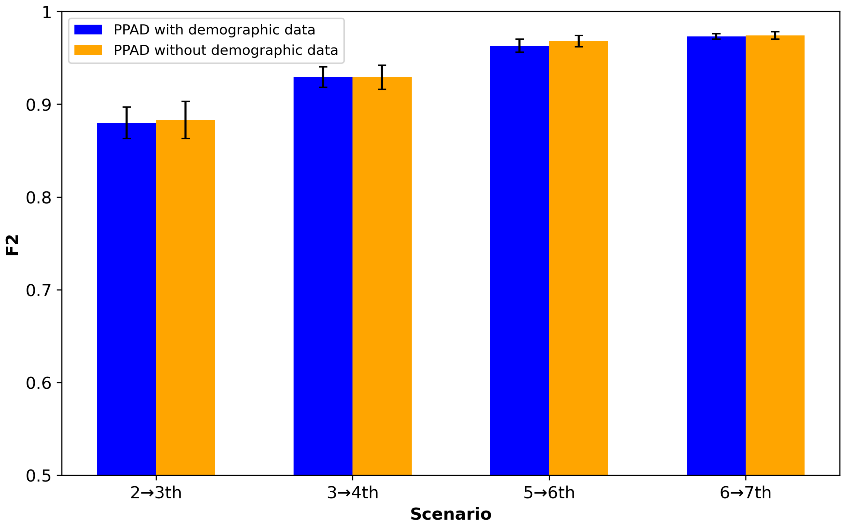

Supplemental Fig. 4 PPAD ablation results for the demographic features. PPAD tested on NACC samples after training using 2, 3, 5, and 6 visits in ADNI.

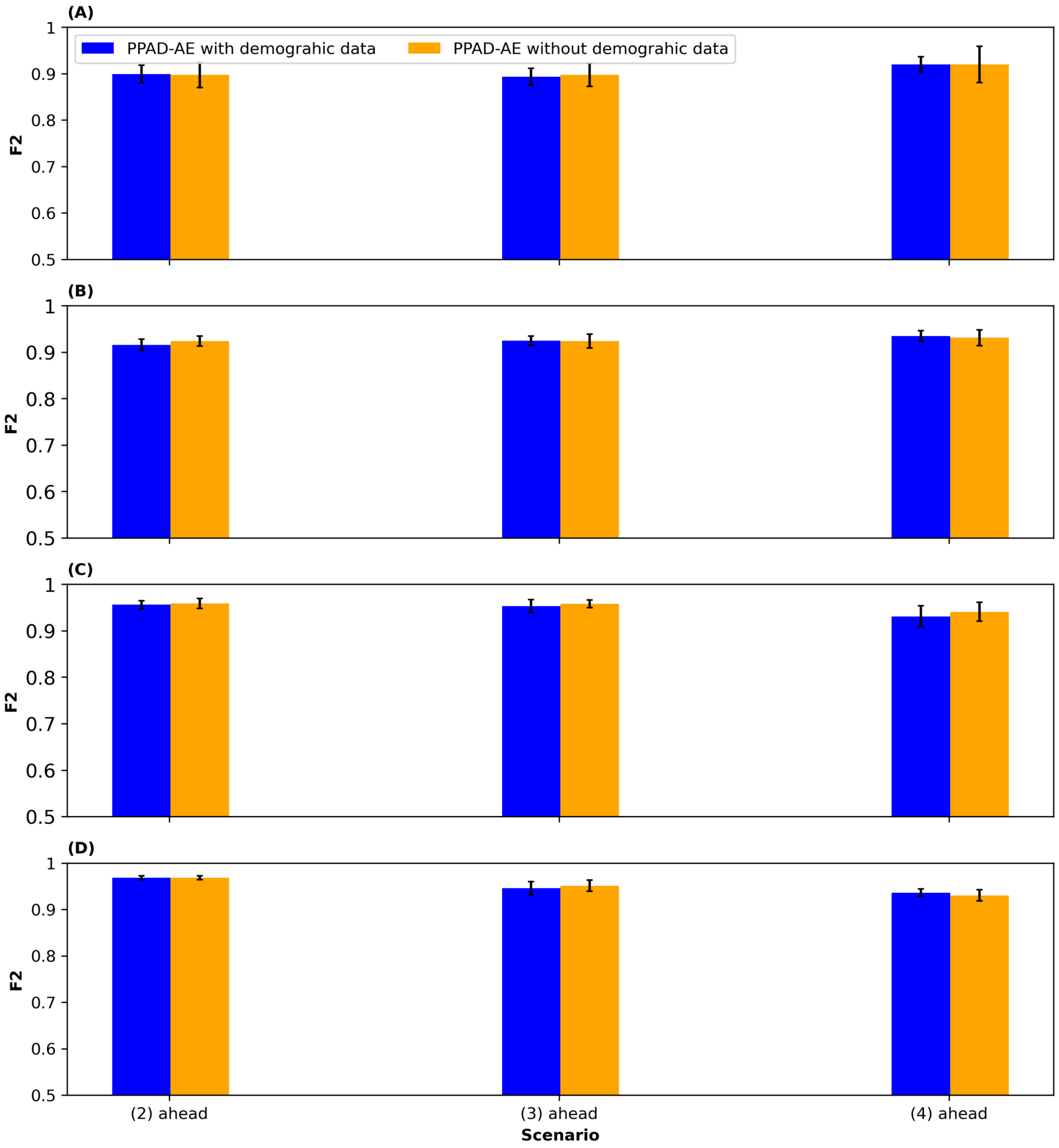

Supplemental Fig. 5 PPAD-AE ablation results for the demographic features. (A, B, C, and D) PPAD-AE tested on NACC samples after training using 2, 3, 5, and 6 visits in ADNI, respectively.
